# Prognostic Significance of Histologic Steatotic Liver Disease in Curatively Resected Non-B, Non-C Hepatocellular Carcinoma

**DOI:** 10.3390/cancers18091447

**Published:** 2026-04-30

**Authors:** Kuan-Hung Wan, Hsin-Ming Wang, Chih-Chi Wang, Yueh-Wei Liu, Wei-Feng Li, Yi-Hao Yen, Yuan-Hung Kuo, Chao-Hung Hung, Tsung-Hui Hu, Wei-Chen Tai, Mu-Jung Tsai, Ming-Chao Tsai

**Affiliations:** 1 Division of Hepato-Gastroenterology, Department of Internal Medicine, Kaohsiung Chang Gung Memorial Hospital and Chang Gung University College of Medicine, Kaohsiung 833401, Taiwan; kevin820813work@gmail.com (K.-H.W.); cassellyen@yahoo.com.tw (Y.-H.Y.); 0104kuo@gmail.com (Y.-H.K.); chh4366@yahoo.com.tw (C.-H.H.); dr.hu@msa.hinet.net (T.-H.H.); luketai1019@gmail.com (W.-C.T.); 2 Kaohsiung Municipal Fong Shan Hospital-Under the Management of Chang Gung Medical Foundation, Kaohsiung 830025, Taiwan; ambulance1027@gmail.com (H.-M.W.); anthony0612@me.com (Y.-W.L.); 3 Division of General Surgery, Department of Surgery, Kaohsiung Chang Gung Memorial Hospital and Chang Gung University College of Medicine, Kaohsiung 833401, Taiwan; ufe14996@ms26.hinet.net (C.-C.W.); webphone0613@yahoo.com.tw (W.-F.L.); 4 School of Medicine, Kaohsiung Medical University Hospital, Kaohsiung 807377, Taiwan; ryan0425@gmail.com; 5 School of Medicine, College of Medicine, National Sun Yat-sen University, Kaohsiung 833201, Taiwan

**Keywords:** hepatocellular carcinoma, metabolic dysfunction-associated steatotic liver disease, non-B non-C, overall survival, steatotic liver disease

## Abstract

**Background/Objectives**: Metabolic dysfunction–associated steatotic liver disease (MASLD) has emerged as a major global etiology of chronic liver disease. However, the prognostic impact of MASLD in patients with non-B, non-C HCC (NBNC-HCC) following curative resection remains poorly defined. This study aimed to evaluate the prognostic significance of histologic SLD and MASLD-related components in this growing patient population. **Methods**: We retrospectively reviewed consecutive patients with NBNC-HCC receiving curative-intent hepatectomy between 2014 and 2023, excluding those with viral hepatitis or significant alcohol use. MASLD was defined as hepatic steatosis (≥5%) combined with at least one cardiometabolic risk factor (obesity, type 2 diabetes, dyslipidemia, or hypertension). Primary endpoints were overall survival (OS) and recurrence-free survival (RFS). Cox proportional hazards models were used to identify independent prognostic factors. **Results**: 169 (61.7%) patients fulfilled MASLD criteria. The MASLD group showed significantly better RFS (*p* = 0.039) and OS (*p* = 0.016). Notably, after multivariate adjustment, histologic SLD remained independently associated with reduced mortality (HR 0.55, 95% CI 0.32–0.93; *p* = 0.027), while MASLD status was attenuated. Subgroup analysis revealed that this survival benefit was most pronounced in non-cirrhotic patients (*p* = 0.027 for OS). Patients with MASLD also exhibited lower liver-related mortality (*p* = 0.028). **Conclusions**: Steatotic liver disease was independently associated with improved survival in NBNC-HCC patients undergoing curative hepatectomy, particularly in non-cirrhotic individuals. Given the increasing prevalence of MASLD, incorporating hepatic steatosis, metabolic components, and fibrosis status into risk stratification may help improve postoperative management in this distinct subgroup.

## Simple Summary

Metabolic dysfunction-associated fatty liver disease is becoming an increasingly important cause of hepatocellular carcinoma, particularly in patients without hepatitis B or hepatitis C infection. However, its influence on outcomes after curative liver resection remains unclear. In this study, we investigated patients with non-viral hepatocellular carcinoma who underwent curative-intent surgery and found that fatty change in the non-tumorous liver was associated with better long-term survival, especially in those without cirrhosis. This favorable association appeared to be more strongly linked to hepatic steatosis itself than to related metabolic conditions, such as diabetes, hypertension, or excess body weight. These findings provide further insight into prognostic stratification after surgery and may help guide postoperative surveillance and management in this growing patient population.

## 1. Introduction

Hepatocellular carcinoma (HCC) remains a major global health burden and is among the leading causes of cancer-related mortality worldwide [1,2]. Although viral hepatitis has historically dominated HCC etiology in Asia and sub-Saharan Africa, an increasing proportion of cases now arise from non-viral causes, paralleling the global rise in metabolic disorders [3,4].

Metabolic dysfunction-associated steatotic liver disease (MASLD) is the most prevalent chronic liver disease worldwide and reflects the close link between hepatic steatosis and cardiometabolic dysfunction [5,6,7]. MASLD increases the risk of advanced fibrosis and cirrhosis and confers excess HCC risk, including in non-cirrhotic livers [8,9,10,11,12]. Given the increasing prevalence of MASLD and its recognized role in hepatocarcinogenesis, understanding its impact on HCC prognosis is of paramount importance [13]. However, the prognostic impact of MASLD after curative-intent resection remains incompletely defined, particularly in patients without chronic hepatitis B or C infection.

Therefore, we investigated the association of histologic SLD, MASLD, and MASLD-related components with recurrence-free survival (RFS) and overall survival (OS) after curative resection in non-viral (NBNC) HCC, aiming to inform postoperative risk stratification and surveillance in this growing population.

## 2. Materials and Methods

This single-center retrospective cohort study was conducted at Kaohsiung Chang Gung Memorial Hospital, Taiwan, and approved by the Institutional Review Board (IRB No.: 202500957B0); informed consent was waived.

### 2.1. Study Population

We identified patients in the institutional HCC registry who underwent curative-intent resection between 2014 and 2023. Patients were excluded if they had received prior HCC treatment, had chronic hepatitis B or C, significant alcohol intake, missing steatosis assessment, prior liver transplantation, or <3 months of follow-up (Figure S1).

### 2.2. Data Collection and Outcomes

Baseline demographics, cardiometabolic comorbidities, laboratory data, and tumor pathology (including microvascular invasion, satellite nodules, differentiation, and cirrhosis) were abstracted at surgery. The primary outcomes were overall survival (OS) and recurrence-free survival (RFS); the secondary outcome was the association of individual MASLD-related components with OS and RFS.

### 2.3. Definition

Steatotic liver disease (SLD) was defined histologically as hepatic steatosis involving ≥5% of hepatocytes in the non-tumorous portion of the resected liver specimen. MASLD was diagnosed based on the presence of SLD, in conjunction with at least one of the following five cardiometabolic risk factors: (1) a BMI ≥ 23 kg/m^2^ for Asian individuals; (2) a prior diagnosis of DM; (3) blood pressure ≥ 130/85 mmHg or current use of antihypertensive medication; (4) plasma triglyceride levels ≥ 150 mg/dL, or treatment with lipid-lowering agents; or (5) low levels of high-density lipoprotein (HDL) cholesterol, defined as <40 mg/dL in males or <50 mg/dL in females, or treatment with lipid-modifying therapy. Patients with alternative causes of hepatic steatosis, particularly excessive alcohol intake (>210 g/week for men and >140 g/week for women), were excluded [14].

HCC was confirmed histologically and defined according to European Association for the Study of the Liver (EASL) and the American Association for the Study of Liver Diseases (AASLD) criteria [15,16]. Tumor staging was performed using the Barcelona Clinic Liver Cancer (BCLC) classification system [17]. Histological grading of tumor differentiation followed the modified nuclear grading method proposed by Edmondson and Steiner, categorizing tumors as well, moderately, or poorly differentiated. Cirrhosis was defined as Ishak fibrosis stage 5–6 in non-tumorous tissue. DM was defined by American Diabetes Association (ADA) criteria [18].

### 2.4. Statistical Analysis

Analyses were performed using SPSS (version 23.0). Continuous variables were expressed as means with standard deviations and compared using Student’s *t*-test or the Mann–Whitney U test, depending on distribution. Categorical variables were analyzed using the chi-square test. Survival was estimated using the Kaplan–Meier method and compared using the log-rank test. Cox proportional hazards models were used for univariable and multivariable analyses. Variables with a *p* value < 0.20 in the univariable Cox regression analysis were considered candidates for inclusion in the multivariable models. Two-sided *p* values < 0.05 were considered statistically significant. Restricted mean survival time (RMST) was calculated to compare survival time between the MASLD/non-MASLD and SLD/non-SLD groups, with truncation time points set at 5 and 10 years.

## 3. Results

### 3.1. Basic Characteristics of Study Subjects

The basic characteristics of the study population are summarized in Table 1. Among 2410 patients who underwent curative resection for HCC, 274 met the inclusion criteria for this analysis. Overall, 177 patients (64.6%) had histologic steatosis (SLD, ≥5%), of whom 169 met MASLD criteria (HCC with MASLD), while the remaining 8 patients had histologic SLD without documented cardiometabolic risk factors (isolated SLD) and were categorized as non-MASLD. The mean age of the entire cohort was 65.5 years, and 68.6% were male. Compared with the non-MASLD group, patients in the MASLD group had a significantly higher body mass index (27.1 vs. 23.4, *p* < 0.001), and a greater prevalence of diabetes mellitus (60.4% vs. 40.0%, *p* = 0.001), hypertension (67.5% vs. 51.4%, *p* = 0.001), and liver cirrhosis (27.8% vs. 16.2%, *p* = 0.027). In terms of liver function, the MASLD group showed higher alanine aminotransferase (ALT) levels (38.9 vs. 28.1 U/L, *p* < 0.001). Histologically, the MASLD group exhibited a higher Ishak fibrosis score. A greater proportion of patients in the MASLD group were also diagnosed at an early stage of HCC (BCLC stage 0/A).

### 3.2. Prognostic Impact of MASLD on Survival and Mortality in NBNC-HCC

**Figure 1:**
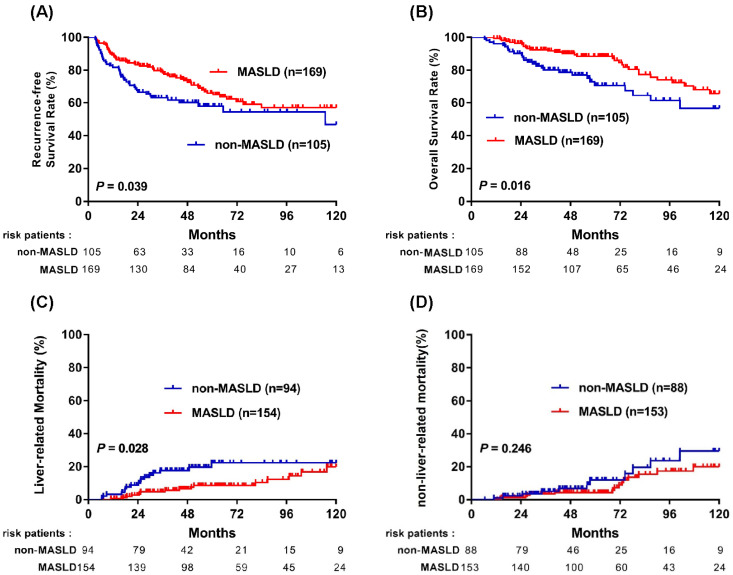
Kaplan–Meier curves comparing survival outcomes between MASLD and non-MASLD groups in patients with NBNC-HCC. (**A**) Recurrence-free survival; (**B**) overall survival; (**C**) liver-related mortality; and (**D**) non-liver-related mortality. Number-at-risk tables are shown below each panel.

As shown in Figure 1, patients with MASLD had significantly better clinical outcomes compared with those without MASLD. Specifically, the MASLD group demonstrated improved recurrence-free survival (RFS) (*p* = 0.039; Figure 1A) and overall survival (OS) (*p* = 0.016; Figure 1B). The Kaplan–Meier-estimated 1-, 3-, and 5-year RFS rates were 92.9%, 83.9%, and 79.1% in the MASLD group, compared to 81.8%, 71.9%, and 63.1% in the non-MASLD group; the corresponding 1-, 3-, and 5-year OS rates were 97.0%, 92.5%, and 90.3% versus 96.2%, 82.6%, and 77.1%, respectively.

In the 5-year RMST analysis, the MASLD group survived an average of 4.69 months longer than the non-MASLD group (56.49 vs. 51.80 months; *p* = 0.010), while the SLD group gained an additional 5.60 months compared to the non-SLD group (56.65 vs. 51.05 months; *p* = 0.003). These survival advantages were even more pronounced in the 10-year evaluation, where the RMST difference for OS was 12.83 months for MASLD versus non-MASLD (102.42 vs. 89.59 months; *p* = 0.020) and 16.97 months for SLD versus non-SLD (103.32 vs. 86.36 months; *p* = 0.003). These findings consistently indicate a statistically significant favorable long-term survival association with steatotic liver disease (Figure S2).

To further evaluate the nature of this survival benefit, causes of death were stratified into liver-related and non-liver-related categories. Liver-related mortality was significantly lower in the MASLD group (*p* = 0.028; Figure 1C), while no statistically significant difference was observed in non-liver-related mortality between the two groups (Figure 1D). Non-liver-related causes of death included cardiovascular events (acute myocardial infarction and heart failure), renal failure, sepsis, and non-HCC malignancies. These findings suggest that MASLD is associated with a reduction in liver-related mortality, which may contribute to the observed improvement in long-term survival.

### 3.3. Steatotic Liver Disease Shows a Stronger Association with Improved Survival than Individual Cardiometabolic Traits

**Figure 2:**
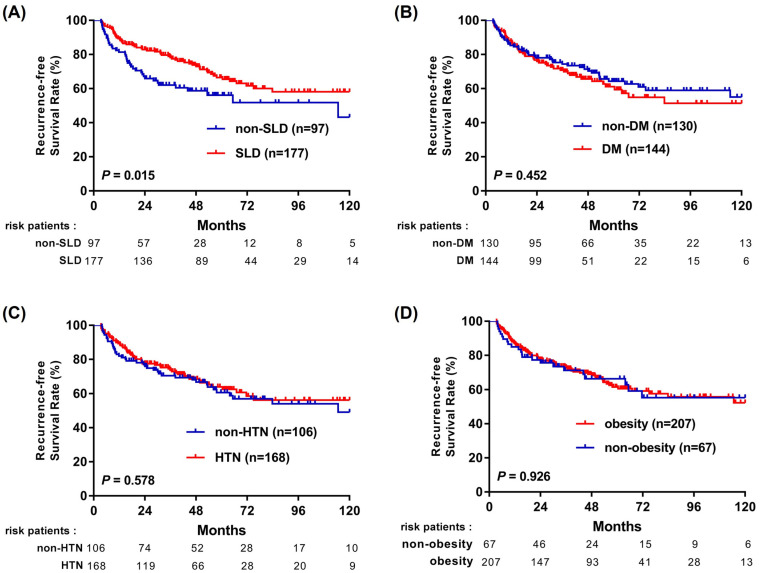
Kaplan–Meier curves for recurrence-free survival in patients with NBNC-HCC stratified according to individual MASLD-related components: (**A**) SLD vs. non-SLD; (**B**) DM vs. non-DM; (**C**) hypertension vs. no hypertension; and (**D**) BMI ≥ 23 vs. <23 kg/m^2^. Number-at-risk tables are shown below each panel.

**Figure 3:**
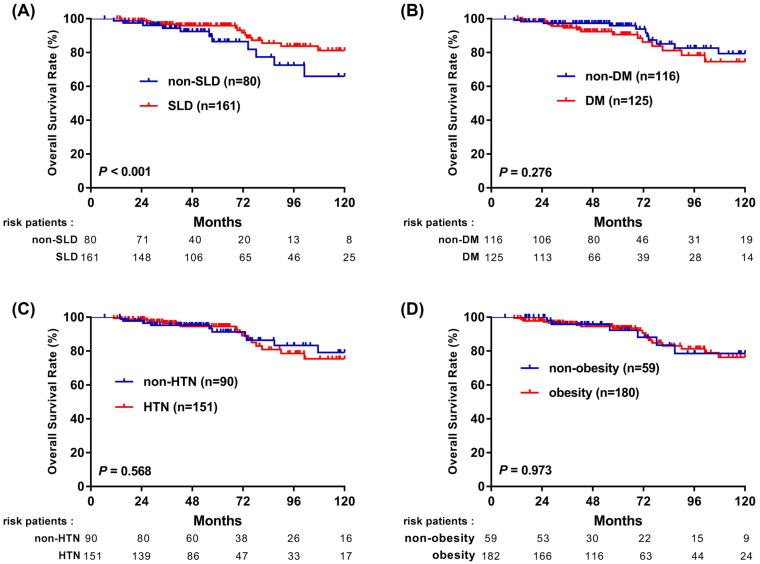
Kaplan–Meier curves for overall survival in patients with NBNC-HCC stratified according to individual MASLD-related components: (**A**) SLD vs. non-SLD; (**B**) DM vs. non-DM; (**C**) hypertension vs. no hypertension; and (**D**) BMI ≥ 23 vs. <23 kg/m^2^. Number-at-risk tables are shown below each panel.

To examine whether the observed survival differences were more closely related to hepatic steatosis or to accompanying cardiometabolic traits, we performed Kaplan–Meier analyses stratified by SLD, DM, HTN, and BMI ≥ 23 kg/m^2^ (Figure 2 and Figure 3). Because these cardiometabolic traits are components of the MASLD definition and are biologically and clinically interrelated, these analyses were interpreted descriptively rather than as assessments of independent causal effects. Among these factors, the presence of SLD showed the strongest association with better outcomes, with improved RFS (*p* = 0.015; Figure 2A) and OS (*p* < 0.001; Figure 3A). In contrast, DM, HTN, and elevated BMI were not significantly associated with either RFS or OS (Figure 2B–D and Figure 3B–D). These findings suggest that the favorable prognosis observed in MASLD patients may be more closely associated with the presence of hepatic steatosis than with individual cardiometabolic traits.

### 3.4. Prognostic Factors Associated with HCC Recurrence

Univariate analysis identified several factors significantly associated with HCC recurrence, including SLD (*p* = 0.016), MASLD (*p* = 0.040) and ALBI grade II (*p* = 0.048) (Table S1). In contrast, diabetes, hypertension, BMI, AFP level, and liver cirrhosis were not significantly associated with recurrence in the univariate model. In the multivariate Cox proportional hazards model, tumor size (hazard ratio [HR], 1.09; 95% confidence interval [CI], 1.05–1.15; *p* < 0.001) and microvascular invasion (MVI) (HR, 1.62; 95% CI, 1.04–2.54; *p* = 0.034) were identified as independent predictors of HCC recurrence. Neither MASLD nor SLD remained significant in the multivariate model.

### 3.5. Factors Associated with All-Cause Mortality in Cox Regression Analysis

Univariate analysis identified older age (*p* = 0.001), larger tumor size (*p* < 0.001), MVI (*p* = 0.001), AFP ≥ 10 ng/mL (*p* = 0.024), and ALBI grade II (*p* < 0.001) as factors associated with an increased risk of all-cause mortality. In contrast, SLD was significantly associated with a reduced risk of all-cause mortality (HR, 0.44; 95% CI, 0.26–0.74; *p* = 0.002), and MASLD showed a similar favorable association (HR, 0.61; 95% CI, 0.38–0.98; *p* = 0.041), whereas diabetes, hypertension, BMI, sex, and liver cirrhosis were not significantly associated with all-cause mortality (Table 2).

In the multivariate Cox proportional hazards analysis, larger tumor size (*p* < 0.001), MVI (*p* < 0.001), older age (*p* = 0.013), and ALBI grade II (*p* < 0.001) were identified as independent predictors of all-cause mortality. Importantly, SLD remained independently associated with a reduced risk of mortality (HR, 0.55; 95% CI, 0.32–0.93; *p* = 0.027), suggesting a potential survival benefit in patients with NBNC-HCC following curative resection.

### 3.6. Subgroup Analysis of MASLD-Associated Survival Outcomes

**Figure 4:**
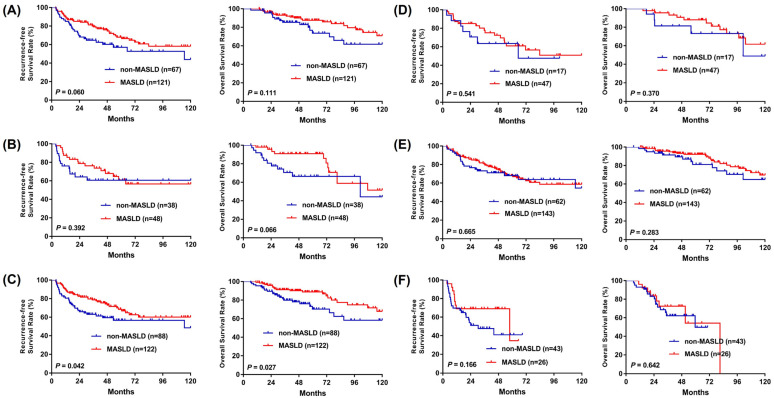
Subgroup analyses of recurrence-free survival and overall survival comparing MASLD and non-MASLD groups in patients with NBNC-HCC. Analyses were stratified by (**A**) male sex, (**B**) female sex, (**C**) absence of liver cirrhosis, (**D**) presence of liver cirrhosis, (**E**) early-stage HCC, and (**F**) advanced-stage HCC.

Subgroup analyses were performed to evaluate the impact of MASLD on RFS and OS across clinically relevant strata in patients with NBNC-HCC (Figure 4). No statistically significant differences in RFS or OS were observed between MASLD and non-MASLD groups when stratified by sex or tumor stage. However, among patients without liver cirrhosis, the MASLD group demonstrated significantly better outcomes, with improved RFS (*p* = 0.042) and OS (*p* = 0.027) compared to their non-MASLD counterparts. These findings suggest that the prognostic benefit associated with MASLD may be more pronounced in patients with preserved hepatic architecture.

### 3.7. Impact of Cardiometabolic Risk Factors on Survival

To investigate the impact of metabolic traits on prognosis, we further stratified patients based on the presence of steatotic liver disease (SLD) and the number of concurrent cardiometabolic risk factors (CMRFs). Notably, all patients in the SLD-only group (0 CMRFs, n = 8) remained alive during the follow-up period, demonstrating the most favorable prognosis. Using the non-SLD group as a reference, the survival advantage remained significant for those with SLD and 1 CMRF (adjusted HR: 0.34, *p* = 0.017) or 2 CMRFs (adjusted HR: 0.49, *p* = 0.047). However, in patients with 3 or more CMRFs, this benefit diminished and lost statistical significance (adjusted HR: 0.53, *p* = 0.054) (Figure S3). These findings suggest a possible attenuation of the survival advantage associated with SLD as the cardiometabolic risk burden increases; however, this exploratory analysis should be interpreted cautiously because of the small subgroup sizes, particularly in the SLD-only group.

## 4. Discussion

In this NBNC-HCC cohort undergoing curative-intent resection, MASLD and SLD were associated with favorable outcomes in unadjusted analyses; however, neither remained independently associated with HCC recurrence after multivariable adjustment. Although MASLD was associated with improved survival in unadjusted analyses, this association was attenuated after adjustment, whereas histologic SLD remained independently associated with overall survival. These findings suggest that the main prognostic signal was related to long-term survival rather than recurrence prevention and appeared to align more closely with histologic steatosis than with MASLD as a composite phenotype. This interpretation supports the prognostic heterogeneity of MASLD, which encompasses a broad spectrum ranging from simple steatosis to steatohepatitis, advanced fibrosis, cirrhosis, and HCC, each associated with distinct levels of oncologic risk and clinical behavior [19].

While numerous studies have established MASLD as a significant risk factor for the development of HCC in the general population [8,9,11,12,13], data specifically evaluating MASLD or SLD as independent prognostic variables after curative resection remain scarce. Most surgical series and pooled analyses have compared NAFLD-/MAFLD-related HCC with viral- or alcohol-related HCC in mixed-etiology cohorts, rather than directly contrasting MASLD/SLD status within NBNC-HCC. Nevertheless, several cohort studies and pooled analyses have suggested that patients with NAFLD-/MAFLD-related HCC may have comparable or even superior post-hepatectomy outcomes relative to those with viral- or alcohol-related HCC, particularly in selected surgical candidates and in Asian populations [20,21,22,23]. In contrast, other studies have reported neutral or unfavorable outcomes in MASLD-related HCC, including a recent systematic review and meta-analysis showing no significant association between MASLD and post-resection survival and a European cohort identifying MASLD as a predictor of poor long-term prognosis after curative surgery [24,25]. These studies provide important context; however, direct comparison should be made cautiously because many prior cohorts included mixed HCC etiologies and/or different treatment modalities, whereas our study specifically focused on patients with NBNC-HCC undergoing curative-intent resection. Differences in study population, etiologic background, treatment setting, background liver disease, and the relative contribution of hepatic steatosis versus metabolic dysfunction may contribute to the heterogeneous prognostic impact of MASLD across studies. To address these uncertainties, we analyzed a homogeneous NBNC-HCC cohort in which MASLD/SLD was explicitly defined as an etiology and patients were stratified according to the presence or absence of MASLD/SLD. In addition, MASLD was further disaggregated into hepatic steatosis and metabolic dysfunction, allowing us to show that MASLD was associated with improved long-term outcomes in unadjusted analyses, whereas the more robust adjusted survival signal was observed for histologic SLD. Consistent with our data, which suggest a favorable prognostic association of SLD and a potential adverse influence of increasing metabolic comorbidity burden (e.g., diabetes, hypertension, obesity), future studies should adopt a more granular approach to risk stratification in MASLD-related HCC. Previous evidence shows that, in patients with chronic hepatitis B and NAFLD, histologic markers of steatohepatitis activity, such as hepatocyte ballooning and advanced fibrosis, independently increase HCC risk [26], further supporting the notion that not all forms or stages of fatty liver disease confer the same prognostic implications.

In our analysis, patients with MASLD exhibited significantly lower liver-related mortality compared to those without MASLD (*p* = 0.028; Figure 1C). This observation suggests that the remnant liver in MASLD patients may experience slower functional deterioration following hepatectomy. Notably, our subgroup analysis further demonstrated that the prognostic benefit of MASLD (for both RFS and OS) was most pronounced in patients without cirrhosis (*p* = 0.042 and *p* = 0.027, respectively; Figure 4C,D), indicating that the favorable association of hepatic steatosis may be especially relevant in the absence of advanced fibrosis. The favorable association between SLD and OS should not be interpreted as evidence of a direct protective biological effect of steatosis itself. Instead, this association may be explained by clinical and host-related factors, including surveillance-driven early detection, preserved hepatic functional reserve, and differences in the clinical course following recurrence. The observation that histologic SLD was independently associated with improved OS, but not with RFS, warrants careful interpretation. This discrepancy suggests that the survival advantage in patients with steatosis is not primarily driven by the prevention of initial tumor recurrence. Rather, the benefit may partly reflect the clinical course following recurrence or other host-related factors. We hypothesize that patients with underlying steatosis may have better preserved liver reserve or clinical conditions that allow them to tolerate repeated sequential therapies—such as re-resection, local ablation, or modern systemic agents—which may contribute to improved OS even when initial recurrence rates are comparable. Additionally, the biological characteristics of recurrent tumors in the context of steatosis may be less aggressive, contributing to a more favorable long-term prognosis. It is important to note, however, that these mechanisms remain hypothetical at this stage. Further granular analysis of post-recurrence treatments, hepatic functional reserve, and tumor biology is required to verify these potential drivers of survival in the SLD population. This pattern aligns with MAFLD-focused reviews indicating that cirrhosis stage is the single strongest determinant of HCC risk in fatty liver disease, whereas patients with mild or no fibrosis have a much lower incidence of HCC and may therefore retain better hepatic reserve and long-term survival potential [19,27]. Consistent with the central role of fibrosis stage in determining prognosis, several surgical series have shown that NAFLD-/MAFLD-related HCC frequently arises in livers with less advanced fibrosis and lower cirrhosis rates than viral hepatitis–related HCC, yet still achieves equal or better long-term survival after resection [20,21].

With the expanding arsenal of therapeutic strategies for hepatocellular carcinoma—including radiofrequency ablation, transarterial chemoembolization, targeted therapies, and immunotherapy—long-term survival is increasingly dependent on a patient’s ability to undergo sequential treatments [28]. This, in turn, is influenced not only by hepatic functional reserve (e.g., Child–Pugh class) but also by overall performance status, nutritional status, and systemic fitness. Whether patients with SLD or MASLD are more likely to maintain stable performance and nutritional reserves after hepatectomy, thereby enabling sustained treatment eligibility, remains an important question for future investigation. The lower liver-related mortality observed in patients with MASLD, together with the independent association between SLD and OS, suggests that these patients may maintain more stable hepatic function post-hepatectomy, which could in turn preserve their eligibility for such sequential therapies upon recurrence. In this context, the favorable long-term overall and recurrence-free survival reported in MAFLD- or NAFLD-related HCC cohorts after resection—despite high rates of metabolic comorbidities—suggests that preserved hepatic reserve and less-severe background liver disease may be key determinants of their capacity to receive repeated locoregional or systemic treatments over time [20,21,22].

In the current study, the survival advantage in MASLD patients was primarily driven by the presence of SLD, whereas individual metabolic components, including diabetes, did not significantly contribute to RFS or OS. While some studies identify diabetes as a risk factor for HCC recurrence, its prognostic impact remains controversial across different surgical cohorts. This lack of statistical significance in our study may stem from cohort heterogeneity, as our population was restricted to patients with NBNC-HCC deemed suitable for curative resection. In such a selected group, the potential adverse effects of metabolic traits may be mitigated by better overall hepatic reserve and closer clinical monitoring. Specifically, we observed that MASLD/SLD patients were diagnosed at a significantly earlier tumor stage despite having more advanced background fibrosis. This suggests that regular surveillance associated with managing metabolic comorbidities facilitates earlier intervention, thereby translating into superior survival outcomes. Thus, the observed prognostic benefit appears to be more a consequence of surveillance-related early detection rather than a direct protective biological effect of the systemic metabolic profile.

Analysis of baseline characteristics revealed significant differences between patients with and without MASLD (Table 1). It is worth noting that while the MASLD/SLD group presented with more advanced background fibrosis and a higher prevalence of cirrhosis, these patients were paradoxically diagnosed at an earlier tumor stage. This apparent paradox may be explained by a combination of biological and clinical mechanisms. Biologically, the clustering of metabolic risk factors—such as diabetes, hypertension, and dyslipidemia—is known to promote persistent hepatic necroinflammation, which can accelerate the progression of fibrosis and increase the risk of cirrhosis in patients with MASLD. Clinically, however, these same comorbidities necessitate more frequent medical encounters for chronic disease management. This increased healthcare contact likely leads to more regular abdominal ultrasonography, facilitating the detection of HCC at a resectable, early stage (BCLC 0/A) through “surveillance-driven early detection.” This lead-time advantage appears to compensate for the more advanced background liver disease, ultimately contributing to the superior survival outcomes observed in this cohort. However, because surveillance intensity and screening intervals were not captured in our retrospective dataset, this explanation remains inferential and warrants confirmation in prospective studies.

Several limitations of this study should be acknowledged. First, the retrospective design inherently introduces a risk of selection bias, despite the application of stringent inclusion and exclusion criteria. Second, the retrospective nature of data collection resulted in missing or incomplete data for certain variables, such as the HOMA-IR index, lipid profiles, and waist circumference, which could have provided deeper insights into the metabolic characteristics of the study cohort. Specifically, because lipid profiles were not routinely available, dyslipidemia could not be incorporated into the MASLD ascertainment process, potentially leading to an under-ascertainment of MASLD. However, this under-ascertainment is likely minimal and expected to have a negligible impact on the overall conclusions of the study. Third, we acknowledge the potential for surveillance bias as a confounding factor. Patients with SLD often have coexisting metabolic traits, such as diabetes or hypertension, which may lead to more frequent medical follow-ups and earlier detection of HCC, potentially contributing to more favorable outcomes. Although propensity score matching was considered to mitigate this bias, it was not performed due to the relatively small cohort size, as matching would have further reduced the statistical power. Fourth, although patients with chronic hepatitis B were excluded based on HBsAg status, the presence of occult hepatitis B infection could not be assessed, as HBV DNA is not routinely tested in HBsAg-negative individuals. Lastly, the relatively small sample size in certain subgroups may have limited the statistical power and generalizability of our subgroup analyses. Future prospective, multicenter studies with more comprehensive metabolic and virologic profiling are needed to validate and expand upon our findings. In addition, we did not systematically assess histologic markers of steatohepatitis activity, such as hepatocyte ballooning and lobular inflammation, which have been shown to independently predict cirrhosis and HCC development in patients with chronic hepatitis B and NAFLD [26]. Incorporating detailed histologic and non-invasive fibrosis assessments—as recommended by recent MAFLD-focused risk stratification frameworks [19]—may further refine prognostic modeling in future NBNC-HCC cohorts.

## 5. Conclusions

In conclusion, our study demonstrates that SLD, rather than MASLD as a whole, was independently associated with improved postoperative overall survival in patients with NBNC-HCC undergoing curative resection, particularly in those without liver cirrhosis. In contrast, neither MASLD nor SLD remained independently associated with HCC recurrence after multivariable adjustment. These findings highlight the prognostic heterogeneity within MASLD and underscore the importance of individualized risk stratification that considers hepatic steatosis and metabolic dysfunction as distinct but related components.

## Figures and Tables

**Table 1:** Characteristics of the 274 patients with non-B, non-C hepatocellular carcinoma with or without metabolic dysfunction-associated steatotic liver disease who underwent curative resection.

	All Patients (n = 274)	HCC with MASLD (n = 169)	HCC Without MASLD (n = 105)	*p* Value
(years), mean ± SD	65.5 ± 11.1	65.0 ± 10.6	66.3 ± 11.9	0.359
Male gender, n (%)	188 (68.6)	121 (71.6)	67 (63.8)	0.177
Body mass index (kg/m^2^), mean ± SD	25.7 ± 4.0	27.1 ± 3.7	23.4 ± 3.4	<0.001
BMI ≥ 23, n (%)	207 (75.5)	153 (90.5)	54 (51.4)	<0.001
DM, n (%)	144 (52.6)	102 (60.4)	42 (40.0)	0.001
Hypertension, n (%)	168 (61.3)	114 (67.5)	54 (51.4)	0.001
Platelet (<150 10^9^/L), n (%)	59 (21.6)	35 (20.7)	24 (23.1)	0.645
AST (U/L), mean ± SD	34.9 ± 19.3	35.9 ± 20.2	33.1 ± 19.5	0.255
ALT (U/L), mean ± SD	34.8 ± 24.2	38.9 ± 25.4	28.1 ± 20.5	<0.001
Total bilirubin (mg/dL), mean ± SD	0.7 ± 0.3	0.7 ± 0.3	0.7 ± 0.3	0.809
Albumin (g/dL), mean ± SD	4.2 ± 0.4	4.2 ± 0.5	4.1 ± 0.4	0.053
ALBI grade, I/II, n (%)	211/60 (77.9/22.1)	136/32 (81/19)	77/28 (72.8/27.2)	0.117
Creatinine (mg/dL), mean ± SD	1.3 ± 0.9	1.1 ± 1.4	1.7 ± 2.4	0.303
AFP (ng/mL), median (range)	9.4 (3.7–112.9)	8.4 (3.6–79.0)	11.6 (3.9–174.5)	0.017
AFP (>10 ng/mL), n (%)	102 (37.2)	62 (36.7)	40 (38.1)	0.815
Ishak score, mean ± SD	2.9 ± 2.0	3.2 ± 1.9	2.4 ± 2.1	0.022
Liver cirrhosis, n (%)	64 (23.4)	47 (27.8)	17 (16.2)	0.027
BCLC stage, n (%)				<0.001
0/A	33/172 (12/62.8)	23/120 (13.6/71.0)	10/52 (9.5/49.5)	
B/C	47/22 (17.2/8.0)	17/9 (10.1/5.3)	30/13 (28.6/12.4)	
Tumor size(cm), mean ± SD	5.0 ± 3.9	4.0 ± 2.5	6.7 ± 5.1	<0.001
Single tumors, n (%)	237 (86.5)	145 (85.8)	92 (87.6)	0.668
Histology grade				0.516
Well, n (%)	44 (16.1)	26 (15.4)	18 (17.1)	
Moderate, n (%)	199 (72.6)	122 (72.2)	77 (73.3)	
Poor, n (%)	28 (10.2)	20 (11.8)	8 (7.6)	
Microvascular invasion, n (%)	90 (32.8)	48 (28.4)	42 (40.0)	0.047
Recurrence, n (%)	97 (35.4)	55 (32.5)	42 (40.0)	0.210
Death, n (%)	59 (21.5)	31 (18.3)	28 (26.7)	0.103
Follow-up (months), mean ± SD	60.6 ± 34.1	65.6 ± 34.1	52.5 ± 32.8	0.002

Data are expressed as mean ± standard deviation (normally distributed), median (interquartile ranges) (not normally distributed) or number (%) accordingly. Abbreviations: BMI, body mass index; DM, diabetes mellitus; AST, aspartate aminotransferase; ALT, alanine aminotransferase; ALBI, Albumin–Bilirubin; AFP, alpha-fetoprotein; BCLC, Barcelona Clinic Liver Cancer.

**Table 2:** Prognostic factors associated with all-cause mortality.

		Univariate		Multivariate	
Variable	Comparison	HR (95% CI)	*p* Value	HR (95% CI)	*p* Value
Age (year)	Per 1 increase	1.04 (1.02–1.07)	0.001	1.03 (1.01–1.06)	0.013
Sex	Male vs. Female	0.64 (0.39–1.05)	0.078		
BMI	≥23 vs. <23	1.14 (0.64–2.03)	0.648		
DM	Yes vs. No	1.42 (0.88–2.29)	0.154		
Hypertension	Yes vs. No	0.93 (0.57–1.49)	0.750		
ALBI grade	II vs. I	2.43 (1.49–3.96)	<0.001	3.11 (1.84–5.21)	<0.001
AFP (ng/mL)	≥10 vs. <10	1.76 (1.08–2.87)	0.024		
Liver cirrhosis	Yes vs. No	0.92 (0.53–1.57)	0.748		
Tumor size (cm)	Per 1 increase	1.20 (1.14–1.20)	<0.001	1.15 (1.09–1.23)	<0.001
Tumor no.	Multiple vs. Single	1.46 (0.78–2.71)	0.238		
Histology stages	Moderate/Poor vs. Well	1.68 (0.77–3.67)	0.195		
MVI	Yes vs. No	2.31 (1.40–3.82)	0.001	2.58 (1.64–4.99)	<0.001
SLD	Yes vs. No	0.44 (0.26–0.74)	0.002	0.55 (0.32–0.93)	0.027
MASLD	Yes vs. No	0.61 (0.38–0.98)	0.041		

Abbreviations: HR, hazard ratio; CI, confidence interval; BMI, body mass index; DM, diabetes mellitus; ALBI, Albumin–Bilirubin; AFP, alpha-fetoprotein; MVI, microvascular invasion; SLD, steatotic liver disease; MASLD, Metabolic dysfunction-associated steatotic liver disease. Note: MASLD was evaluated in a separate multivariable model to avoid collinearity with SLD, but it did not reach statistical significance.

## Data Availability

The data that support the findings of this study are not publicly available due to privacy and ethical restrictions.

## Supplementary Materials

The following supporting information can be downloaded at https://www.mdpi.com/article/10.3390/cancers18091447/s1, Figure S1: Patient selection flowchart; Figure S2: Restricted mean survival time analyses; Figure S3: Survival according to SLD status and number of cardiometabolic risk factors; Table S1: Prognostic factors associated with hepatocellular carcinoma recurrence.

## Abbreviations

The following abbreviations are used in this manuscript:

MASLD	Metabolic dysfunction-associated steatotic liver disease
HCC	hepatocellular carcinoma
NBNC-HCC	non-B, non-C hepatocellular carcinoma
OS	overall survival
RFS	recurrence-free survival
HR	hazard ratio
CI	confidence interval
NBNC	non-B, non-C
IRB	Institutional Review Board
SLD	steatotic liver disease
BMI	body mass index
DM	diabetes mellitus
HDL	high-density lipoprotein
EASL	European Association for the Study of the Liver
AASLD	American Association for the Study of Liver Diseases
BCLC	Barcelona Clinic Liver Cancer
ADA	American Diabetes Association
SPSS	Statistical Package for the Social Sciences
RMST	restricted mean survival time
ALT	alanine aminotransferase
HTN	hypertension
ALBI	albumin–bilirubin
AFP	alpha-fetoprotein
MVI	microvascular invasion
CMRFs	cardiometabolic risk factors
MAFLD	metabolic dysfunction-associated fatty liver disease
NAFLD	nonalcoholic fatty liver disease
HOMA-IR	Homeostasis Model Assessment of Insulin Resistance
HBsAg	hepatitis B surface antigen
HBV DNA	hepatitis B virus DNA
AST	aspartate aminotransferase
