# Supplementary material for: Prognostic Significance of Histologic Steatotic Liver Disease in Curatively Resected Non-B, Non-C Hepatocellular Carcinoma
Source: Cancers (Basel). 2026 Apr 30;18(9):1447. doi: 10.3390/cancers18091447 (PMC13163080; doi:10.3390/cancers18091447)
Supplement: Supplementary file 1 [file cancers-18-01447-s001.zip › Supplementary Table.pdf]

Table S1. Prognostic factors associated with hepatocellular carcinoma recurrence

		Univariate		Multivariate	
**Variable**	**Comparison**	**HR (95%CI)**	***p* value**	**HR (95%CI)**	***p* value**
Age(year)	Per 1 increase	1.02 (0.99-1.04)	0.062		
Sex	Male vs Female	0.85 (0.56-1.31)	0.467		
BMI	≥ 23 vs < 23	0.98 (0.61-1.56)	0.926		
DM	Yes vs No	1.17 (0.78-1.74)	0.453		
Hypertension	Yes vs No	0.89 (0.59-1.34)	0.579		
ALBI grade	II vs I	1.57 (1.01-2.45)	0.048		
AFP (ng/mL)	≥10 vs < 10	1.26 (0.83-1.93)	0.283		
Liver cirrhosis	Yes vs No	1.03 (0.65-1.62)	0.900		
Tumor size(cm)	Per 1 increase	1.12 (1.07-1.16)	<0.001	1.09 (1.05-1.15)	<0.001
Tumor no.	Multiple vs Single	1.39 (0.82-2.38)	0.225		
Histology stages	Moderate/Poor vs Well	1.17 (0.67-2.06)	0.584		
MVI	Yes vs No	2.11 (1.40-3.17)	<0.001	1.62 (1.04-2.54)	0.034
SLD	Yes vs No	0.61 (0.40-0.91)	0.016		
MASLD	Yes vs No	0.66 (0.44-0.98)	0.040		

Abbreviations: HR, hazard ratio; CI, confidence interval; BMI, body mass index; DM, diabetes mellitus; ALBI, Albumin-Bilirubin; AFP, alpha fetoprotein; BCLC, Barcelona clinic liver cancer; MVI, microvascular invasion; SLD, steatotic liver disease; MASLD, Metabolic dysfunction-associated steatotic liver disease
