# Supplementary figures and images for: Prognostic Significance of Histologic Steatotic Liver Disease in Curatively Resected Non-B, Non-C Hepatocellular Carcinoma
Source: Cancers (Basel). 2026 Apr 30;18(9):1447. doi: 10.3390/cancers18091447 (PMC13163080; doi:10.3390/cancers18091447)

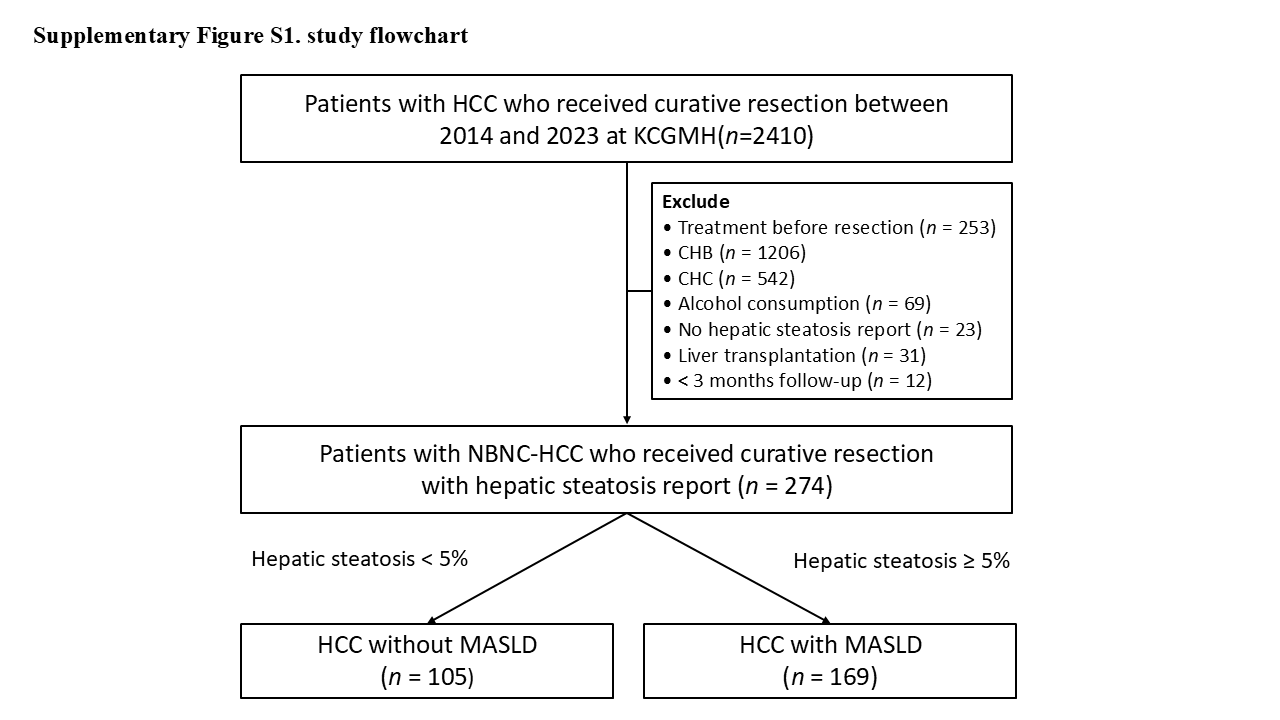

Supplement: Supplementary file 1 [file cancers-18-01447-s001.zip › Supplementary Figure S1.tiff]

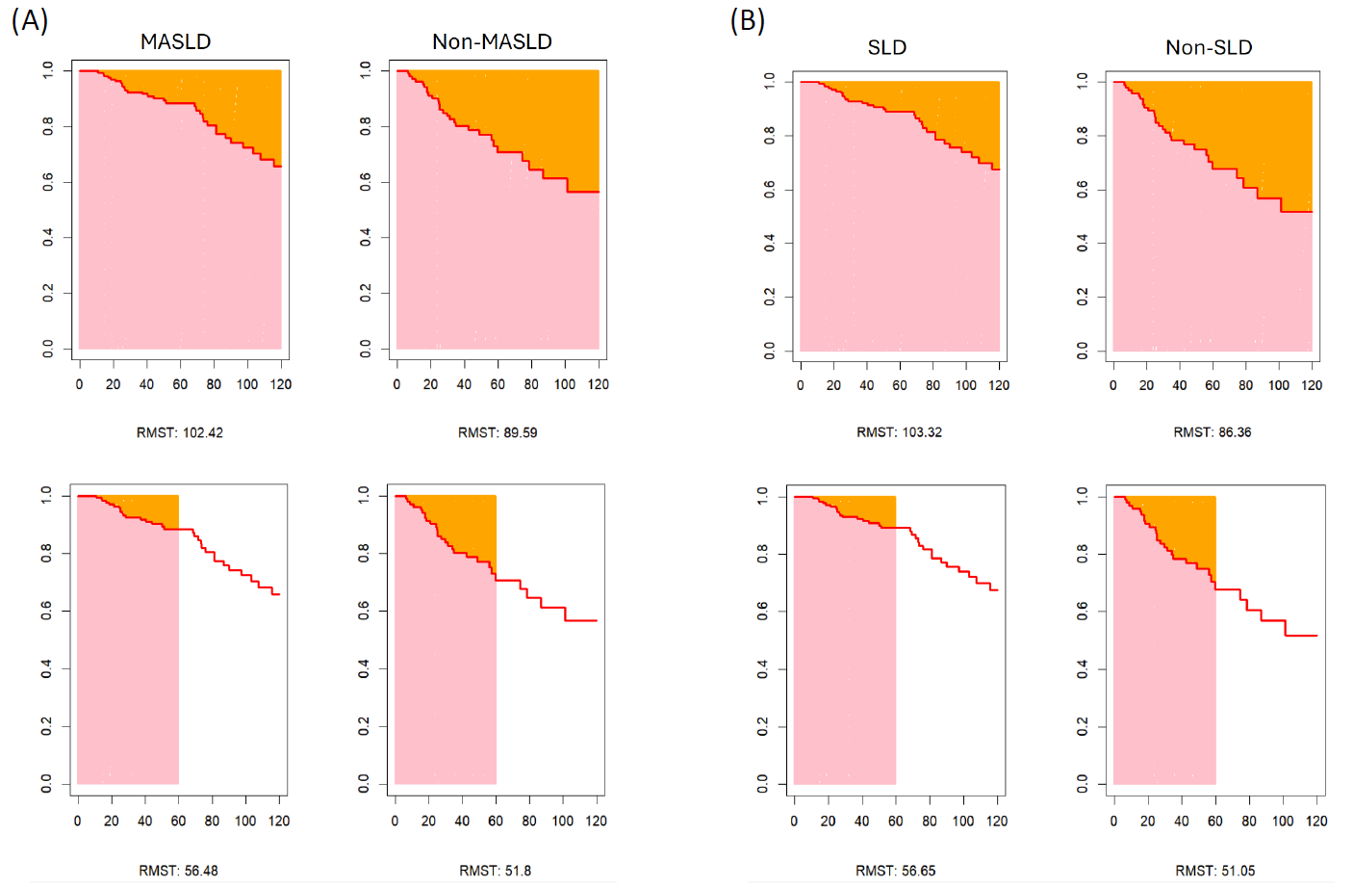

Supplement: Supplementary file 1 [file cancers-18-01447-s001.zip › Supplementary Figure S2.tif]

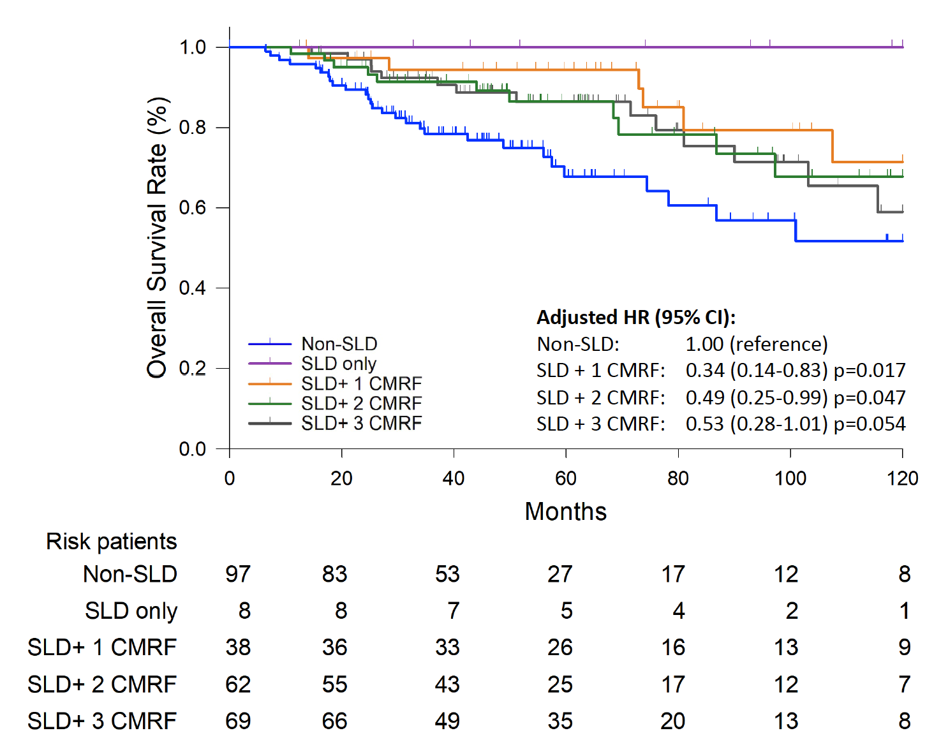

Supplement: Supplementary file 1 [file cancers-18-01447-s001.zip › Supplementary Figure S3.tif]
